# New Perspectives in the Medical Treatment of Non-Muscle-Invasive Bladder Cancer: Immune Checkpoint Inhibitors and Beyond

**DOI:** 10.3390/cells11030357

**Published:** 2022-01-21

**Authors:** Alessandro Audisio, Consuelo Buttigliero, Marco Donatello Delcuratolo, Elena Parlagreco, Marco Audisio, Antonio Ungaro, Rosario Francesco Di Stefano, Lavinia Di Prima, Fabio Turco, Marcello Tucci

**Affiliations:** 1 Department of Oncology, University of Turin, San Luigi Gonzaga Hospital, Orbassano, 10093 Turin, Italy; alessandro.audisio93@gmail.com (A.A.); donatello.m.delcuratolo@gmail.com (M.D.D.); elena.parlagreco@edu.unito.it (E.P.); marco.audix@gmail.com (M.A.); antonio.unga@gmail.com (A.U.); rosario-distefano@virgilio.it (R.F.D.S.); lavinia.diprima@unito.it (L.D.P.); turcofabio9@gmail.com (F.T.); 2 Department of Medical Oncology, Cardinal Massaia Hospital, 14100 Asti, Italy; marcello.tucci@gmail.com

**Keywords:** non-muscle-invasive bladder cancer, BGC-unresponsive, immunotherapy, immune-checkpoint inhibitors, pembrolizumab

## Abstract

Non-muscle-invasive bladder cancer (NMIBC) is characterized by a high rate of cure, but also by a non-negligible probability of recurrence and risk progression to muscle-invasive disease. NMIBC management requires a proper local resection and staging, followed by a risk-based treatment with intravesical agents. For many years, the current gold standard treatment for patients with intermediate or high-risk disease is transurethral resection of the bladder (TURB) followed by intravesical bacillus Calmette–Guérin (BCG) instillations. Unfortunately, in about half of high-risk patients, intravesical BCG treatment fails and NMIBC persists or recurs early. While radical cystectomy remains the gold standard for these patients, new therapeutic targets are being individuated and studied. Radical cystectomy in fact can provide an excellent long-term disease control, but can deeply interfere with quality of life. In particular, the enhanced immune checkpoints expression shown in BCG-unresponsive patients and the activity of immune checkpoints inhibitors (ICIs) in advanced bladder cancer provided the rationale for testing ICIs in NMIBC. Recently, pembrolizumab has shown promising activity in BCG-unresponsive NMIBC patients, obtaining FDA approval. Meanwhile multiple novel drugs with alternative mechanisms of action have proven to be safe and effective in NMIBC treatment and others are under investigation. The aim of this review is to analyse and describe the clinical activity of new emerging drugs in BCG-unresponsive NMIBC focusing on immunotherapy results.

## 1. Introduction

Bladder cancer (BC) represents the tenth most common cancer worldwide and the fifth diagnosed in high-income countries [1]. It has been found that three-quarters of BC patients present at the diagnosis with Non-Muscle-Invasive Bladder Cancer (NMIBC), a localized disease restricted to the mucosa (Ta or carcinoma *in situ*, CIS, according to AJCC/TNM staging of bladder cancer) or invading the *lamina propria* (T1). The remaining patients have a muscle-invasive bladder cancer (MIBC) or a metastatic BC, with poorer prognosis [2,3]. Cancer-specific 5-year survival is estimated to be more than 90% in NMIBC, this percentage drops to 50% to 82% in MIBC treated with radical cystectomy (RC), while only a small percentage of metastatic BC patients is alive at 5 years from diagnosis [4].

NMIBC management is characterized for a major part of patients by a locoregional intravesical approach. A diagnostic–therapeutic cystoscopy with transurethral resection of the bladder (TURB) is indicated in case of Ta or T1 tumours and multiple bladder biopsies are recommended to detect CIS [5,6]. Further treatment should be tailored to the risk of recurrence and/or progression into MIBC. International guidelines recommend a clinical–pathological classification of NMIBC into low-, intermediate-, and high-risk groups [5,6,7]. Low-risk NMIBC (primary, low-grade Ta or T1 tumour, without CIS) do not usually require any further treatment after TURB and a single immediate intravesical instillation of chemotherapy, usually mitomycin C (MMC). Patients with high grade Ta or T1 are included in intermediate or high-risk group and the current gold standard treatment is TURB followed by six-weekly induction of intravesical bacillus Calmette–Guérin (BCG) instillation followed by maintenance [7]. CIS is a high-grade tumour with a more than 50% chance of disease progression [8]. In case of CIS presence, patients are included in the high-risk group. Standard treatment is intravesical BCG instillation with a higher complete response (CR) rates and a longer duration of response compared to intravesical chemotherapy [9].

However, in about half of patients, intravesical BCG treatment fails and high-risk NMIBC persists, in case of BCG-refractory tumour, or recurs after a CR, in case of BCG-relapsing tumour [10]. BCG-unresponsive NMIBC includes both patients with BCG-refractory tumours and early BCG-relapsing tumours (within six months of their last BCG instillation) and has the highest risk of disease progression. An additional category, defined as BCG-intolerant NMIBC, includes patients with disease recurrence after a suboptimal therapy due to a BCG-related adverse events [2].

BCG-unresponsive tumours do not benefit from further BCG instillations and current guidelines recommend early RC with urinary diversion as a preferred option [5,6,7]. This major surgical procedure can provide an excellent long-term disease control, but can negatively impact on quality of life and not all patients are eligible for or accept this procedure [11,12]. Non-surgical alternatives until recently have been ineffective and their benefit is rather to postpone the BC than to achieve a long-term disease control. The intravesical administration of valrubicin, an anthracycline, is the only medical treatment approved by Food and Drug Administration (FDA) with a CR rate of 21%, mostly temporary [13].

Several other intravesical chemotherapeutic agents have been investigated as gemcitabine, docetaxel, nanoparticle albumin-bound (nab)-paclitaxel, the sequential instillation of gemcitabine and docetaxel, or MMC, obtaining suboptimal and not lasting response rates [14,15,16,17,18]. Recently, immunotherapy with pembrolizumab, an anti-Programmed cell Death protein 1 (PD1) monoclonal antibody (mAb), has demonstrated promising antitumor activity in BCG-unresponsive NMIBC in the phase II trial KEYNOTE-057 obtaining FDA approval [19]. Meanwhile, several novel agents with different mechanisms have proven to be safe and effective in the treatment of NMIBC (Table 1) and more agents are currently under investigation.

The aim of this review is to describe the clinical activity of new emerging drugs in BCG-unresponsive NMIBC, focusing on immune checkpoint inhibitors (ICIs).

## 2. Immunotherapy in NMIBC: From BCG to the New Horizons of ICIs

### 2.1. BCG Administration Drives an Antitumour Innate and Adaptative Immune Response in NMIBC

**Figure 1:**
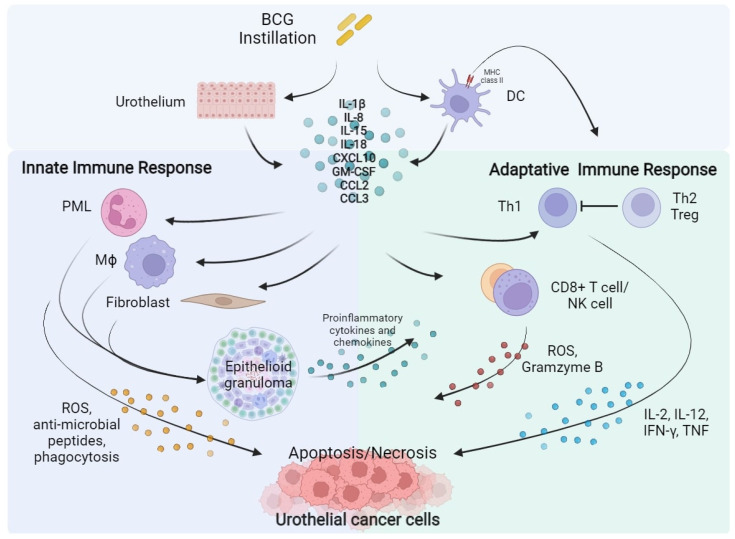
BCG instillation elicits both innate and adaptative immune response against urothelial cancer cells. BCG, Bacillus Calmette–Guérin; DC, Dendritic Cell; IL, Interleukin; CXCL10, C-X-C motif Chemokine Ligand 10; GM-CSF, Granulocyte-Macrophage Colony-Stimulating Factor; CCL2, C-C Motif Chemokine Ligand 2; CCL3, C-C Motif Chemokine Ligand 3; PML, Polymorphonuclear Leukocytes; Th, Helper T cell; Treg, Regulatory T cell; Mϕ, Macrophage; CD8, Cluster of Differentiation 8; NK, Natural Killer; ROS, Reactive Oxygen Species; IFN-γ, Interferon-γ; TNF, Tumour Necrosis Factor.

BCG is a live attenuated strain of *Mycobacterium bovis*, its activity on NMIBC was firstly demonstrated by Morales and colleagues in 1976 [23]. The BCG mechanism of action is still not completely understood; however, it is known that BCG exposure of urothelium and bladder-resident macrophages elicits an inflammatory and immune response against tumoral cells [24,25,26]. The presumed mechanism is explicated in Figure 1.

The activation of antigen-presenting cells (APC) and urothelial cells following BCG internalization induces the release of several cytokines as Interleukin (IL)-1b, IL-8, IL-15, IL-18 and chemokine as CXC motif chemokine ligand 10 (CXCL10), Granulocyte-Macrophage Colony-Stimulating Factor (GM-CSF), CC-motif Chemokine ligand 2 (CCL2), and CCL3 activating the innate and adaptive immune response [27]. Local innate immunity activation leads to recruitment of macrophages, granulocytes, fibroblasts, dendritic cells, and lymphocytes, which form typical epithelioid and gigantocellular granulomas. In addition, neutrophils, Cluster of differentiation 8 (CD8) + T cells, and Natural Killer (NK) cells may have a direct antitumour effect, inducing the production of reactive oxygen species (ROS), antimicrobial enzymes and pro-apoptotic factors [28,29,30]. Concurrently, BCG causes the expression of the major histocompatibility complex (MHC) class II presenting BCG antigens on APC and urothelial cells driving the activation of adaptative immunity [31,32]. A prominent T Helper (TH) 1 cells-mediated immune response, associated with the secretion of IL-2, IL-12, Interferon (IFN)-γ, and Tumour Necrosis Factor (TNF) correlates with a response to BCG instillation. On the contrary, TH2 cells activation, characterized by the releasing of IL-4, IL-5, IL-6, and IL-10, is associated with an immunosuppressive microenvironment enrich of T regulatory cells (Treg), which is associated to a BCG-unresponsive state [24,33].

Several randomized controlled studies and large meta-analysis have clearly demonstrated that intravesical BCG after TURB, administered with an induction schedule of 6 weekly instillations, followed by additional maintenance every 3 to 6 months over 1 to 3 years is significantly superior compared to TURB alone or TURB followed by intravesical chemotherapy in NMIBC recurrence prevention. BCG treatment provides an high rate of CR in both patients with high-risk papillary tumours and with CIS and lowers tumour progression risk, representing the standard treatment for these patients [34].

### 2.2. PD-L1 and PD-1 Expression Is Associated to BCG Immune-Resistance

The main resistance mechanism to BCG treatment is linked to an intrinsic or an acquired immune resistance. The interaction of PD-1, expressed by T cells, with its ligand Programmed Death-Ligand 1 (PD-L1), normally expressed by a subset of macrophages and inducible on activated T, B and NK cells, endothelial cells, and other non-malignant cells in an inflammatory milieu is a major immune checkpoint pathway involved in immune homeostasis, down-regulating T cell response in case of chronic antigen exposure. Cancer-related overexpression of PD-L1 lets cancer cells to evade immune response, inducing T cell anergy. The use of PD-1 or PD-L1-directed mAb, can prevent their interaction and restore T cell activity against cancer cells [35,36].

Kates and colleagues showed in an analysis on tissue microarrays of paired pre- and post-BCG bladder samples that BCG-unresponders patients had in 25–30% of cases a pre-treatment enrichment of PD-L1 + cells, high density of CD8+ T cells, and lacked of CD4+ T cells. On the contrary, PD-L1 expression was nearly absent among BCG responders [37]. Pierconti et al. confirmed these results. PD-L1 expression in tumour cells and in immune cells was higher in BCG-unresponsive CIS patients than in BCG-responders, suggesting that PD-L1 expression could help to identify CIS that would fail BCG therapy [38]. In addition, BCG treatment could enhance PD-L1 and PD-1 expression. Hashizume et al. observed that PD-L1 expression levels increased after BCG. Similarly, Fukumoto et al., testing PD-1 staining in a cohort of NMIBC treated with BCG, found that PD-1 expression was superior in BCG-unresponsive tumors compared with pretreatment tumors from the same patients, hypothesizing that BCG could induce this immune checkpoint. Furthermore, PD-1 expression was correlated with worse clinical outcomes [39,40]. BCG instillation seems to induce the expression of PD-L1 in tumour and inflammatory cells trough the induction of CD8+ T cells, which are the major responsible of IFN-γ production [39]. Chevalier and colleagues reported an increasing number of PD-L1-expressing CD4+ T cells (PD-L1+ Tregs) in BCG-resistant patients [41], while Copland et al. demonstrated that BCG treatment causes the up-regulation of PD-L1 expression on APCs inducing the secretion of some cytokines as Il-6, IL-10, leading to STAT3 phosphorylation and ultimately PD-L1 expression [42].

### 2.3. ICIs for the Treatment of Advanced Urothelial Cancer

The high tumoral mutational burden (TMB) of urothelial cancer, similar to melanoma and non-small-cell lung cancer, and the expression of immune checkpoint PD-1 and PD-L1 both by immune cells and microenvironmental cells constitute the biological rational for the activity of ICIs in bladder cancer [43,44]. Nowadays ICIs represent the standard second-line therapy in patients with advanced or metastatic urothelial cancer who progressed on first-line platinum-based chemotherapy. Pembrolizumab, an anti-PD-1 mAb, according to results of phase III trial KEYNOTE-045 is the preferred option [45,46]. First-line immunotherapy does not provide a statistical significant survival benefit compared to platinum-based chemotherapy, even when it was given in association; however, avelumab, an anti-PD-L1 monoclonal IgG, as maintenance in patients who did not have disease progression with first-line chemotherapy, gets the approval on the basis of JAVELIN Bladder 100 [46,47]. Moreover, nivolumab, another PD-1 mAb was recently granted FDA approval for the adjuvant treatment of patients with urothelial carcinoma who are at high risk of recurrence after RC on the basis of results of CheckMate-274, and several trials are investigating the role of ICIs in neoadjuvant and perioperative setting [46,48]. Clinical or biological markers predictive of response are still lacking; however, PD-L1 expression and elevated TMB status seem to be correlated with an increased response rate [49].

### 2.4. ICIs Activity in BCG-Unresponsive NMIBC

The enhanced immune checkpoint expression shown in BCG-unresponsive patients and the efficacy of ICIs in advanced BC represented the rationale for testing them in NMIBC. Pembrolizumab was investigated in the phase II KEYNOTE-057 trial (Table 1). In the cohort A of the study, intravenous pembrolizumab was administered for up to 24 months in patients with BCG-unresponsive CIS patients, who resulted ineligible or declines RC. After a median follow-up of 36.4 months, 41% of patients (95% CI 30.7–51.1%) achieved a CR assessed by cystoscopy and urine cytology. Eleven of 39 patients with CR (28%) were disease-free at data cut-off analysis. Results of the study cohort B, which enrolled patients with BCG-unresponsive NMIBC without CIS, have not been published yet. Safety profile was consistent with other studies testing pembrolizumab; serious treatment-related adverse events (G3 or G4 according to World Health Organization, WHO) were rare [19]. On the basis of these results, in January 2020, FDA approved pembrolizumab for the treatment of patients with BCG-unresponsive CIS who are ineligible for or who decline RC [19].

Atezolizumab was tested in the phase II SWOG S1605 trial (Table 1). One hundred and thirty-five patients with BCG-unresponsive NMIBC were enrolled, 70 of them had been diagnosed with CIS, and atezolizumab was given them every 3 weeks up to complete one year of treatment. Thirty patients had a CR at 3 months (41.1%; 95% CI 29.7–53.2%) and 19 at 6 months (26.0%; 95% CI 16.5–37.6%) [50]. In the overall population, 29 patients (29%; 90% CI 22–36%) were free of recurrence or progression at 18 months, the percentage of event-free survival was greater in non-CIS patients than in CIS patients. The treatment was globally well tolerated. Serious grade adverse events occurred in 17% of patients and there were two treatment-related deaths [20].

Several clinical trials are now ongoing testing different ICIs in BCG-unresponsive NMIBC (Table 2). Durvalumab, an anti-PD-L1 Immunoglobulin G1 (IgG1) mAb, camrelizumab, an anti PD-1 ICI, and HX008 (pucotenlimab), a new recombinant anti-PD-1 monoclonal IgG4 are being tested as monotherapy respectively in NCT04738630, NCT04706598, NCT03759496. ADAPT-BLADDER study (NCT03317158) is investigating durvalumab activity in association with radiotherapy, while PREVERT trial (NCT03950362) activity of avelumab. Durvalumab is, furthermore, being evaluated in association with an anti-CTLA4 mAb, tremelimumab in RIDEAU study (NCT05120622). SunRISe-1 study (NCT04640623) endpoints are to assess the efficacy and safety of TAR-200, an intravesical gemcitabine-delivery system, in association with an anti PD-1 mAb, cetrelimab, or of these two drugs alone in BCG-unresponsive high-risk NMIBC. NCT04164082 trial investigates the combination of pembrolizumab and gemcitabine.

As explicated before, BCG-resistance could be linked to an immunosuppressive state induced by the expression of immune checkpoint, and BCG itself could enhance PD-1 and PD-L1; this provides the grounds for trials that are testing anti-PD1 or anti-PD-L1 antibodies in association with BCG as front-line therapy in NMIBC in BCG-naïve patients or in patients not reaching a CR after BCG induction (Table 2). KEYNOTE-676 (NCT03711032) is a phase III trial assessing pembrolizumab activity in combination with BCG in patients with persistent high-risk NMIBC after BCG induction. CheckMate 7G8 (NCT04149574) and POTOMAC (NCT03528694) studies testing respectively nivolumab and durvalumab have a similar design. NCT03892642 is a phase I/II trial planned to evaluate BCG in association with avelumab as induction treatment. The primary endpoint of the phase I of the trial was the completion of a full induction course. The primary endpoint was reached, the combination of BCG with an ICI was reported to be safe and well tolerated, and phase II is still ongoing [51]. The NCT04730232 study is testing tislelizumab in association with nab-paclitaxel chemotherapy, while in the DURANCE trial (NCT04106115), it is in combination with S-488210/S-488211, a cancer multi-peptide vaccine able to stimulate a cytotoxic T cell (CTL) response against urothelial cancer cells [52].

## 3. Alternative Targets: The Way to Develop New Effective Drugs

**Figure 2:**
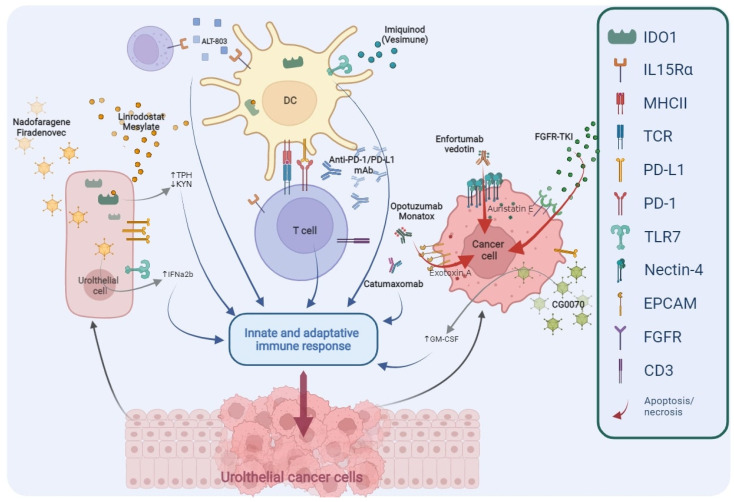
Main targets of novel drugs being investigated in BCG-unresponsive NMIBC. TPH, Tryptophan; KYN, Kynurenine; IFNα2b, Interferon α2b; DC, Dendritic Cell; PD1, Programmed cell Death protein 1; PD-L1, Programmed Death-Ligand 1; mAb, monoclonal Antibody; GM-CSF, Granulocyte-Macrophage Colony-Stimulating Factor; FGFR, Fibroblast Growth Factor Receptor; TKI, Tyrosine Kinase Inhibitors; IDO1, Indoleamine 2,3-Dioxygenase 1; IL-15Rα, Interleukin-15 receptor α; MHCII, Major Histocompatibility Complex Class II; TCR, T Cell Receptor; TLR7, Toll-like Receptor 7; EpCAM, Epithelial Cell Adhesion Molecule; CD3, Cluster of Differentiation 3.

The deep improvement in the knowledge regarding the biological mechanisms responsible for neoplastic cells progression and BCG resistance mechanisms has led to identification of new targets (Figure 2); consequently, several innovative agents were developed and are now under investigation in the treatment of NMIBC (Table 3).

### 3.1. Emerging Immune Modulators in NMIBC

#### 3.1.1. Nadofaragene Firadenovec

IFN-α is a cytokine able to block tumour cell growth directly inducing apoptosis, and indirectly through angiogenesis inhibition and stimulation of both innate and adaptive immune response against bladder cancer cells [53]. Intravesical instillation of IFN-α2b demonstrated to be well tolerated and to have a dose-related clinical activity; however, the duration of response is poor due to the rapid voiding elimination [54]. Nadofaragene firadenovec (rAd-IFNa/Syn3) is an intravesical adenovirus vector-based gene therapy that carries human recombinant IFN-α2b gene into the bladder epithelium, allowing a constant IFN-α2b intravesical concentration [55]. Intravesical nadofaragene firadenovec was evaluated in a phase I and in a phase II study enrolling BCG-unresponsive NMIBC, proving to be safe and active [56,57]. The results of these studies provided the basis for the phase III trial. In this study, 53.4% (95% CI 43.3–63.3%) of BCG-unresponsive NMIBC patients with CIS had a CR at 3-months evaluation and 24.3% of patients had a CR lasting more than one year. In the cohort of patients without CIS, 35 of 48 patients (72.9%; 95% CI 58.2–84.7%) were recurrence free at 3 months, and 21 patients (43.8%; 95% CI 29.5–58.8%) at 12 months (Table 1). The trial confirmed the drug good tolerability, most frequent adverse events were transient, bladder-related and classified as either grade 1 or 2, with no new concerns [21]. At the extended follow-up analysis, with a mean of 23.5 months, 30 of the 55 patients achieving a CR remained recurrence free; the reported cystectomy-free survival was 69.8% (95% CI 54.3–80.9%) [58,59].

#### 3.1.2. CG0070

CG0070 is a conditionally replicating oncolytic adenovirus which selectively replicates in retinoblastoma (Rb) pathway–defective bladder tumour cells, which carry the coding DNA for human GM-CSF. Two mechanisms of action are recognised: the direct antineoplastic activity, killing the host cells, in which it replicates, and the indirect activity, inducing a specific, long-lasting antitumor immune response trough GM-CSF [60]. In a phase II trial, 45 of 66 BCG-unresponsive NMIBC patients treated with CG0070 completed a 6-months follow up. Overall, 6-month CR was 47% (95% CI 32–62%), 58% in the CIS group and 33% in the Ta/T1 group. Treatment proved to be safe and treatment–related adverse events were most commonly bladder-related [61]. CG0070 is currently under investigation as monotherapy in the phase III registration trial (BOND-003, NCT04452591) and in association with pembrolizumab in a phase II trial (CORE-001, NCT04387461). Both trials are enrolling BCG-unresponsive NMIBC.

#### 3.1.3. BMS-986205 (Linrodostat Mesylate)

Indoleamine-2,3-dioxygenase 1 (IDO1) is an enzyme involved in the conversion of tryptophan to kynurenine and has a key immunoregulatory role. Tryptophan starvation, reducing the capacity of protein synthesis, induces anergy of T cells, while the production of kynurenine, trough the activation of aryl-hydrocarbon receptor, causes an immunosuppressive state regulated by IL-10 and Treg cells [62]. Several IDO1-inhibitors are currently under investigation in different BC setting. Linrodostat mesylate (BMS-986205), a selective and potent oral IDO1-inhibitor, is being studied in association with nivolumab with or without BCG in BCG-unresponsive NMIBC in CheckMate 9UT trial (NCT03519256) [63].

#### 3.1.4. ALT-803

IL-15 receptor α is crucial for recruitment and activation of effector NK cells and CD8+ T cells [64]. Preclinical evidence showed that a potent IL-15 receptor-agonist, ALT-803, could stimulate both adaptive and innate immune response, showing anti-tumoral activity in bladder cancer models [65]. In a phase I trial, nine patients with BCG-naïve NMIBC were treated with BCG in association with ALT-803. Twenty-four months after treatment start all patients were disease-free and no severe adverse events were reported [66]. The second part phase II of this trial is currently ongoing (NCT02138734). NCT03022825 is a phase II/III trial testing BCG and ALT-803 in BCG-unresponsive NMIBC. Preliminary results seem to confirm the high activity of this combination, nine out of eleven patients (82%) with CIS demonstrated a CR [67].

#### 3.1.5. Other Immune Modulators

Toll-like receptors (TLRs) are the key receptors of innate immune response. These receptors are present on immune cells, bladder cells, and even on neoplastic cells. The interaction of TLR with their ligands, defined as pathogen-associated molecular patterns (PAMPs), stimulates immune response; BCG itself, through its antigens, can act on TLR, in particular on TLR2 and TLR4 [68]. Imiquimod is a TLR7 agonist and in a preclinical model it has a boost effect on BCG [69]. A novel liquid formulation optimized for intravesical delivery, Vesimune (TMX-101), is being investigated in a phase II study. Preliminary results seem to confirm its immunological activity, inducing an increase of urinary cytokines as IL-6 and IL-8; however, clinical data have not been published yet [70].

VPM1002BC is a recombinant Mycobacterium bovis BCG strain able to improve immunogenicity. Safety and activity of VPM1002BC in NMIBC recurrence after conventional BCG therapy were tested in the phase I/II trial (NCT02371447). This treatment proved to be active with a recurrence-free survival at 60 weeks after the first instillation of 49.3% (95% CI 32.1%, 64.4%) and at the same time well tolerated [71].

Finally, a cancer vaccine composed of a combination of the injectable formulations of S-488210 and S-488211 is currently under investigation in association with durvalumab in the DURANCE trial (NCT04106115).

### 3.2. Targeting Adhesion Molecules: EpCAM and Nectin-4

Adhesion molecules expressed by tumoral cells, as Epithelial Cell Adhesion Molecule (EpCAM) or Nectin-4, are other important focus of research.

EpCAM is an intercellular adhesion molecule, with a crucial role in cells differentiation and proliferation. It is highly expressed in a variety of epithelial neoplasia, being directly involved in tumourigenesis [72].

Oportuzumab monatox is an antibody–drug conjugate (ADC)-like recombinant fusion protein of humanized anti-EpCAM antibody and Pseudomonas exotoxin A administered intravesically. The antibody-component binds to the cancer cell causing the internalization of the molecule. Once internalized it releases the cytotoxic exotoxin A, which induces apoptosis. In a phase II trial 40% of BCG-unresponsive CIS patients had a CR, 16% of patients had a CR that was maintained at 1 year-evaluation [73]. In a phase III single-arm trial, the CR rate in CIS patients was 40% with a medium duration of response of 9.4 months (95% CI 5.1 months-not reached) (Table 1). In the subgroup of patients achieving a response after 3 months, more than half were recurrence-free at 12 months [22]. Treatment with oportuzumab monatox was well tolerated, the most frequent adverse events were local bladder-related symptoms [22,73].

Catumaxomab is a bispecific antibody targeting EpCAM and T-cell antigen CD3. According to this dual mechanism of action, EpCAM-positive cancer cells are highly efficiently killed by the immune cells. First data on clinical use of intravesical catumaxomab in BCG-unresponsive NMBIC suggest that it is feasible, safe, and efficacious. Currently, a phase I/II trial is ongoing (NCT04799847).

Nectin-4 is an Ig superfamily member, its primary role is maintaining adherent junctions together with cadherin. Nectin-4 has been associated with tumourigenesis, promoting cancer cell proliferation, and it is overexpressed in most urothelial carcinomas. Enfortumab Vedotin is an ADC, consisted in an anti-Nectin-4 antibody linked to auristatin E, a cytotoxic microtubule-disrupting agent. The efficacy of Enfortumab Vedotin in metastatic or advanced bladder cancer has been demonstrated and this activity granted the FDA approval [74]. A feasibility phase I study enrolling BCG-unresponsive NMIBC is starting (NCT05014139).

### 3.3. FGFR: A Common Altered Gene in Early-Stage BC

Fibroblast growth factor receptors (FGFRs) are a family of 5 different type 1 transmembrane tyrosine kinase receptors, which transduce signal in response to extracellular stimuli. Activating mutations of FGFR are wide common in NMIBC and they can be up to 75% in low-grade papillary tumours; conversely, they are less frequent in MIBC [75]. Several FGFR-tyrosine kinase inhibitors (TKIs) are currently under investigation in multiple therapeutic setting of BC, including NMIBC. A phase II trial aiming to assess the clinical and pharmacodynamic activity of dovitinib, an oral FGFR/Vascular Endothelial Growth Factor Receptor (VEGFR) inhibitor in patients with BCG-refractory NMIBC with FGFR3 mutation or over-expression failed to meet its primary endpoint, with a 6-month CR rate of only 8% [76]. Three other trials are currently ongoing in patients with BCG-refractory NMIBC. Two studies are testing erdafitinib, a pan-FGFR TKI (NCT04917809, NCT04172675) and one pemigatinib, a FGFR1-3 inhibitor (NCT03914794).

### 3.4. Other Agents with Peculiar Pharmacodynamic Activity

Alternative drugs with specific mechanisms of action are also being tested. APL-1202 is a selective Methionine Aminopeptidase 2 (MetAP2) inhibitor. MetAP2 is an intracellular metalloprotease crucial in tumourigenesis and tumour microenvironment homeostasis. MetAP-2 inhibition could have a combined effect on endothelial cells, with an antiangiogenetic effect, and on tumour cells [77]. NCT04498702 is a phase II trial testing APL1202 in BCG-unresponsive NMIBC; preliminary results of the safety and activity on Ta or T1 tumour have been published. This treatment was demonstrated to be safe, and one-year recurrence free rate was reported to be 54.3% (95% CI 37.2–73.2%) [78]. APL-1202 activity is also studied in BCG-naïve patients (NCT04736394).

Finally, while FGFR3 mutation occurs mostly in low-grade NMIBC, gain-of-function mutations within the Phosphoinositide 3-kinases (PI3K)/AKT/mammalian target of rapamycin (mTOR) pathway are most common in high-grade NMIBC [79]. A phase I/II of a nab- version of rapamycin, Nab-sirolimus, in BCG-refractory demonstrated its safety and activity. The results of the study testing the combination of intravesical gemcitabine and nab-sirolimus have not been published yet (NCT02009332).

## 4. Alternative Ways to Deliver Chemotherapy: Chemo-Hyperthermia and EMDA-MMC

Intravesical chemotherapy has limited activity and provides mostly temporary responses in BCG-unresponsive NMIBC. Device-assisted treatments with the aim of improving the activity of intravesical chemotherapy have been developed and they are currently under investigation. Their common underlying mechanism is to increase the permeability of the bladder wall to chemotherapy; this can be achieved by several means [80].

Chemohyperthermia consists of the intravesical administration of chemotherapy heated up to more than 40° using an extra-corporeal heating system or an intravesical microwave applicator. In three prospective single-arm trials, these procedures proved to be safe and effective in prevent disease recurrence and progression of BCG unresponsive NMIBC, mostly of low- or intermediate-risk. However, data on high-risk BCG-unresponsive NMIBC, with or without CIS, are scarce [81,82,83].

Another technique is the Electromotive Drug Administration (EMDA)-MMC, which exploits the phenomenon called “iontophoresis”, created by intravesical electrodes, to improve the absorption of MMC. In a phase II trial enrolling patients with BCG-unresponsive NMIBC, EMDA-MMC demonstrated to have only a modest activity in CIS patients, while this system seemed to be effective in preventing the recurrence of papillary tumours (Ta or T1) [84].

## 5. Conclusions

Radical cystectomy with pelvic lymph nodes dissection and urinary diversion still represents the backbone of BCG-unresponsive NMIBC treatment, but this surgical procedure can negatively affect patients’ quality of life. For this reason, many patients decline this procedure. Moreover, some patients are ineligible to surgery because of frailty or comorbidities. For more than 20 years, only valrubicin had FDA approval for the treatment of BCG-unresponsive CIS.

BCG-resistance could be mediated by immune checkpoints expression by immune cells, cancer cells, and tumoural microenvironment and the PD-1/PD-L1 pathway is among the best known. Anti-PD-1 monoclonal antibody pembrolizumab was the first to demonstrate, in the KEYNOTE-057 trial, that in patients who cannot be eligible due to comorbidities or refusing RC, systemic immunotherapy could be safe and effective in preventing or at least delaying the need of surgery. Pembrolizumab is the first immunotherapeutic agent to gain FDA approval for the treatment of patients with BCG-unresponsive CIS, with or without papillary tumours, who are ineligible for or refuse to undergo RC. In addition, the preliminary results of the SWOG S1605 trial confirm that an anti-PD-L1 monoclonal antibody, atezolizumab, can be safe and active. Driven by these findings, ICIs are now being tested in BCG-naïve setting, mostly in association with BCG instillation, in order to improve the response rate and reduce disease recurrence.

Nowadays, other prosing pharmacodynamics mechanism have been explored; intravesical nadofaragene firadenovec, a recombinant IFN-α2b gene delivered into urothelium by adenovirus vector, and intravesical oportuzumab monatox, an ADC of anti-EpCAM antibody conjunct to Pseudomonas exotoxin A, have already proven to have an efficacy comparable to pembrolizumab, and they could become part of the drug armamentarium available soon. Meanwhile, other agents, as oncolytic virus, immune modulators as IDO1 inhibitors or IL-15 agonist, antibodies targeting intercellular junctions, FGFR-TKIs, are still being tested.

## Figures and Tables

**Table 1:** Key positive clinical trials enrolling patients with BCG-unresponsive NMIBC.

Agent/ Target	NCT/ Acronym	Phase	Primary Endpoint	Patients Enrolled	Median Follow Up	Results
**Pembrolizumab** * ICI Anti-PD1 IgG4/kappa	NCT02625961 KEYNOTE-057 [19]	II	CRR of high-risk NMIBC	Cohort A (CIS): 101 pts Cohort B (Non-CIS): 47 pts	36.4 mos.	Cohort A: 41% (39 out of 96 pts, 95% CI 30.7–51.1%)
**Atezolizumab** ICI Anti-PD-L1 IgG1	NCT02844816 SWOG S1605 [20]	II	CRR at 25 weeks in CIS-cohort	CIS cohort: 70 pts pre-planned Non-CIS cohort: 65 pts pre-planned	NR	CIS cohort: 27% (20 out of 74 pts, 95% CI NR)
**Nadofaragene firadenovec** rAd-IFNa2b/Syn3	NCT02773849 [21]	III	CRR at 12 mos. in CIS-cohort	CIS-cohort: 107 pts Non-CIS cohort: 50 pts	19.7 mos.	CIS-cohort: 53.4% (55 out of 103 patients, 95% CI 43.3–63.3%)
**Oportuzumab Monatox** EpCAM scFv linked to ETA	NCT02449239 [22]	III	CRR in CIS-cohort	126 pts CIS-cohort: 89 pts	NR	CIS-cohort: 40% (95% CI NR)

* FDA approved. NCT, Number Clinical Trial; ICI, Immune Checkpoint Inhibitor; PD1, Programmed cell Death protein-1; IgG, Immunoglobulin G; CRR, Complete Response Rate; NMIBC, Non-Muscle-Invasive Bladder Cancer; CIS, Carcinoma *In Situ*; mos., months; pts, patients; PD-L1, Programmed death-Ligand 1; NR, Not reported; rAd-IFNa2b, non-replicating recombinant adenovirus type 5 (Ad5)-vector encoding the interferon alpha-2b; EpCAM, Epithelial Cell Adhesion Molecule; scFv, single-chain Fragment variable; ETA, Pseudomonas exotoxin A; BCG, Bacillus Calmette–Guérin.

**Table 2:** Ongoing clinical trials testing ICIs (in bold font) alone or in combination in NMIBC.

NCT/Acronym	Status	Phase	Drug(s)	Control	Primary Endpoints
**(a) BCG-unresponsive or BCG-intolerant NMIBC**					
NCT05120622 Rideau	Recruiting	1, 2	**Durvalumab, tremelimumab**	—	TRAEs, MTD
NCT04738630	Recruiting	2	**HX008** (Pucotenlimab)	—	CRR, EFS
NCT04706598	Recruiting	1, 2	**Camrelizumab**	—	MTD, RFS
NCT04640623 SunRISe-1	Recruiting	2	TAR-200, **Cetrelimab**	TAR-200 or Cetrelimab	CRR
NCT04387461 CORE-001	Recruiting	2	CG0070, **Pembrolizumab**	—	CRR
NCT04164082	Recruiting	2	**Pembrolizumab**, gemcitabine	—	CRR in CIS subpopulation, EFS
NCT03950362 PREVERT	Not yet recruiting	2	**Avelumab**, RDT	—	RFS
NCT03759496	Recruiting	2	**Durvalumab**	—	MTD, RFS
NCT03519256 CheckMate 9UT	Active, not recruiting	2	**Nivolumab**, BMS-986205 (Linrodostat mesylate)	Nivolumab	CRR, DoR
NCT03317158 ADAPT-BLADDER	Recruiting	1, 2	**Durvalumab**, RDT	—	RP2D, RFS
NCT04149574 CheckMate 7G8	Recruiting	3	**Nivolumab**, BCG	BCG	EFS
NCT04106115 DURANCE	Not yet recruiting	1, 2	**Durvalumab**, S-488210/S-488211 vaccine	—	DLT, DFSR
NCT03892642 ABC Trial	Active, not recruiting	1, 2	**Avelumab**, BCG	—	DLT
**(b) BCG-naïve NMIBC**					
NCT04922047 TACBIN-01	Recruiting	1, 2	**Tislelizumab**, BCG	—	DLT
NCT04730232	Recruiting	2	**Tislelizumab**, nab-paclitaxel	—	CRR
NCT04165317 * CREST	Recruiting	3	**Sasanlimab**, BCG	BCG	EFS, CRR
NCT03799835 ALBAN	Recruiting	3	**Atezolizumab**, 1y BCG	BCG	RFS
NCT03711032 * KEYNOTE-676	Recruiting	3	**Pembrolizumab**, BCG	BCG	CRR, EFS
NCT03528694 POTOMAC	Active, not recruiting	3	**Durvalumab**, BCG	BCG	DFS

(a) Enrolling patients with BCG-unresponsive or BCG-intolerant NMIBC. (b) Enrolling patients with BCG-naïve NMIBC. * Enrolling patient with either BCG-unresponsive or BCG-naïve NMIBC. NCT, Number Clinical Trial; BCG, Bacillus Calmette–Guérin; NMIBC, Non-Muscle-Invasive Bladder Cancer; TRAEs, Treatment-Related Adverse Events; MTD, Maximum Tolerated Dose; CRR, Complete Response Rate; EFS, Event-Free Survival; RFS, Recurrence-Free Survival; CIS, Carcinoma *In Situ*; RP2D, Recommended phase 2 dose; DLT, Dose-Limiting Toxicity.

**Table 3:** Clinical trials testing novel or emerging drugs (in bold font) alone or in combination in NMIBC.

NCT/Acronym	Status	Phase	Drug(s)	Target or Mechanism	Primary Endpoints
**(a) BCG-unresponsive or BCG-intolerant NMIBC**					
NCT05014139	Not yet recruiting	1	**Enfortumab Vedotin**	ADC against Nectin-4	TRAEs, DLT
NCT04917809	Not yet recruiting	2	**Erdafitinib**	FGFR-TKI	ORR
NCT04799847	Not yet recruiting	1, 2	**Catumaxomab**	Bispecific (anti-EpCAM, anti-CD3) Ab	DLT, TRAEs
NCT04498702	Completed	2	**APL-1202**	MetAP2 inhibitor	RFR
NCT04452591 BOND-003	Recruiting	3	**CG0070**	Oncolytic adenovirus	CRR
NCT04172675	Recruiting	2	**Erdafitinib** vs. gemcitabine/MMC	FGFR-TKI	RFS
NCT03914794	Recruiting	2	**Pemigatinib**	FGFR1-3-TKI	CRR
NCT03022825 QUILT-3.032	Recruiting	2, 3	BCG, **ALT-803**	IL-15 superagonist	CRR, DFR
NCT02009332	Completed	1, 2	**Nab-sirolimus**, gemcitabine	mTOR inhibitor	DLT, CRR
NCT01731652	Completed	2	**Vesimune**	TLR-7 agonist	CRR
NCT02371447	Active, not recruiting	1, 2	**VPM1002BC**	Modified BCG	DLT, RFR
**(b) BCG-naïve NMIBC**					
NCT04736394 ASCERTAIN	Not yet recruiting	3	**APL-1202** vs. epirubicin	MetAP2 inhibitor	EFS
NCT02138734	Recruiting	1, 2	**ALT-803**, BCG	IL-15 superagonist	CRR, DFS

(a) Enrolling patients with BCG-unresponsive or BCG-intolerant NMIBC. (b) Enrolling patients with BCG-naïve NMIBC. NCT, Number Clinical Trial; BCG, Bacillus Calmette–Guérin; NMIBC, Non-Muscle-Invasive Bladder Cancer; ADC, Antibody-Drug Conjugate; TRAEs, treatment-related Adverse Events; DLT, Dose-Limiting Toxicity; MMC, Mitomycin C; FGFR, Fibroblast Growth Factor-receptor; TKI, Tyrosine Kinase Inhibitor; ORR, Objective Response Rate; EpCAM, Epithelial Cell Adhesion Molecule; CD3, Cluster of Differentiation 3; Ab, Antibody; MetAP2, Methionyl Aminopeptidase 2; RFR, Recurrence-free Rate; CRR, Complete Response Rate; RFS, Recurrence-Free Survival; IL-15, Interleukin-15; DFR, Disease-Free Rate; mTOR, Mammalian Target of Rapamycin; TLR-7, Toll-Like Receptor 7; EFS, Event-Free Survival.

## References

[1] Globocan IARC Cancer Today (2020). Bladder Cancer Fact Sheet. https://gco.iarc.fr/today/data/factsheets/cancers/30-Bladder-fact-sheet.pdf.

[2] Compérat E., Gontero P., Mostafid A.H., Palou J., Van Rhijn B.W.G., Rouprêt M., Shariat S.F., Sylvester R., Zigeuner R. (2019). Non-muscle-invasive Bladder Cancer (TaT1 and CIS) EAU Guidelines. Eur. Urol..

[3] Kamat A.M., Hahn N.M., Efstathiou J.A., Lerner S.P., Malmström P.U., Choi W., Guo C.C., Lotan Y., Kassouf W. (2016). Bladder cancer. Lancet.

[4] Powles T., Bellmunt J., Comperat E., De Santis M., Huddart R., Loriot Y., Necchi A., Valderrama B.P., Ravaud A., Shariat S.F. (2021). Bladder cancer: ESMO clinical practice guideline for diagnosis, treatment and follow-up. Ann. Oncol..

[5] Babjuk M., Burger M., Capoun O., Cohen D., Compérat E.M., Dominguez Escrig J.L., Gontero P., Liedberg F., Masson-Lecomte A., Mostafid A.H. (2021). European Association of Urology Guidelines on Non–muscle-invasive Bladder Cancer (Ta, T1, and Carcinoma in Situ). Eur. Urol..

[6] National Comprehensive Cancer Network Bladder Cancer. NCCN Clin Pract Guidel Oncol. https://www.nccn.org/professionals/physician_gls/pdf/bladder.pdf.

[7] Chang S.S., Boorjian S.A., Chou R., Clark P.E., Daneshmand S., Konety B.R., Pruthi R., Quale D.Z., Ritch C.R., Seigne J.D. (2016). Diagnosis and Treatment of Non-Muscle Invasive Bladder Cancer: AUA/SUO Guideline. J. Urol..

[8] Lamm D.L. (1992). Carcinoma in situ. Urol. Clin. N. Am..

[9] Sylvester R.J., van der Meijden A.P.M., Witjes J.A., Kurth K. (2005). Bacillus calmette-guerin versus chemotherapy for the intravesical treatment of patients with carcinoma in situ of the bladder: A meta-analysis of the published results of randomized clinical trials. J. Urol..

[10] Sfakianos J.P., Kim P.H., Hakimi A.A., Herr H.W. (2014). The effect of restaging transurethral resection on recurrence and progression rates in patients with nonmuscle invasive bladder cancer treated with intravesical bacillus Calmette-Guérin. J. Urol..

[11] See W.A. (2007). Postoperative nomogram predicting risk of recurrence after radical cystectomy for bladder cancer: International Bladder Cancer Nomogram Consortium, Bochner BH, Kattan MW, Vora KC, Department of Urology, Memorial Sloan-Kettering Cancer Center, Kimmel Center for Prostate and Urologic Tumors, New York, NY. Urol. Oncol. Semin. Orig. Investig..

[12] Cambier S., Sylvester R.J., Collette L., Gontero P., Brausi M.A., Van Andel G., Kirkels W.J., Da Silva F.C., Oosterlinck W., Prescott S. (2016). EORTC Nomograms and Risk Groups for Predicting Recurrence, Progression, and Disease-specific and Overall Survival in Non-Muscle-invasive Stage Ta-T1 Urothelial Bladder Cancer Patients Treated with 1-3 Years of Maintenance Bacillus Calmette-Guérin. Eur. Urol..

[13] Steinberg G., Bahnson R., Brosman S., Middleton R., Wajsman Z., Wehle M. (2000). Efficacy and safety of valrubicin for the treatment of Bacillus Calmette-Guerin refractory carcinoma in situ of the bladder. The Valrubicin Study Group. J. Urol..

[14] Dalbagni G., Russo P., Bochner B., Ben-Porat L., Sheinfeld J., Sogani P., Donat M.S., Herr H.W., Bajorin D. (2006). Phase II trial of intravesical gemcitabine in bacille Calmette-Guérin-refractory transitional cell carcinoma of the bladder. J. Clin. Oncol..

[15] Barlow L.J., McKiernan J.M., Benson M.C. (2013). Long-term survival outcomes with intravesical docetaxel for recurrent nonmuscle invasive bladder cancer after previous bacillus Calmette-Guérin therapy. J. Urol..

[16] McKiernan J.M., Holder D.D., Ghandour R.A., Barlow L.J., Ahn J.J., Kates M., Badalato G.M., Roychoudhury A., Decastro G.J., Benson M.C. (2014). Phase II trial of intravesical nanoparticle albumin bound paclitaxel for the treatment of nonmuscle invasive urothelial carcinoma of the bladder after bacillus Calmette-Guérin treatment failure. J. Urol..

[17] Steinberg R.L., Thomas L.J., O’Donnell M.A., Nepple K.G. (2015). Sequential Intravesical Gemcitabine and Docetaxel for the Salvage Treatment of Non-Muscle Invasive Bladder Cancer. Bladder Cancer.

[18] Lightfoot A.J., Breyer B.N., Rosevear H.M., Erickson B.A., Konety B.R., O’Donnell M.A. (2014). Multi-institutional analysis of sequential intravesical gemcitabine and mitomycin C chemotherapy for non-muscle invasive bladder cancer. Urol. Oncol..

[19] Balar A.V., Kamat A.M., Kulkarni G.S., Uchio E.M., Boormans J.L., Roumiguié M., Krieger L.E.M., Singer E.A., Bajorin D.F., Grivas P. (2021). Pembrolizumab monotherapy for the treatment of high-risk non-muscle-invasive bladder cancer unresponsive to BCG (KEYNOTE-057): An open-label, single-arm, multicentre, phase 2 study. Lancet Oncol..

[20] Black P.C., Tangen C., Singh P., McConkey D.J., Lucia S., Lowrance W.T., Koshkin V.S., Stratton K.L., Bivalacqua T., Kassouf W. (2021). Phase II trial of atezolizumab in BCG-unresponsive non-muscle invasive bladder cancer: SWOG S1605 (NCT #02844816). J. Clin. Oncol..

[21] Boorjian S.A., Alemozaffar M., Konety B.R., Shore N.D., Gomella L.G., Kamat A.M., Bivalacqua T.J., Montgomery J.S., Lerner S.P., Busby J.E. (2021). Intravesical nadofaragene firadenovec gene therapy for BCG-unresponsive non-muscle-invasive bladder cancer: A single-arm, open-label, repeat-dose clinical trial. Lancet Oncol..

[22] Shore N., O’Donnell M., Keane T., Jewett M.A., Kulkarni G.S., Dickstein R., Wolk F., Dunshee C., Belkoff L., Dillon R.L. (2020). PD03-02 Phase 3 results of Vicineum in BCG-unresponsive Non-Muscle Invasive Bladder Cancer. J. Urol..

[23] Morales A., Eidinger D., Bruce A.W. (1976). Intracavitary Bacillus Calmette-Guerin in the treatment of superficial bladder tumors. J. Urol..

[24] Larsen E.S., Joensen U.N., Poulsen A.M., Goletti D., Johansen I.S. (2020). Bacillus Calmette–Guérin immunotherapy for bladder cancer: A review of immunological aspects, clinical effects and BCG infections. Apmis.

[25] Ingersoll M.A., Albert M.L. (2013). From infection to immunotherapy: Host immune responses to bacteria at the bladder mucosa. Mucosal Immunol..

[26] Teppema J.S., de Boer E.C., Steerenberg P.A., van der Meijden A.P. (1992). Morphological aspects of the interaction of Bacillus Calmette-Guérin with urothelial bladder cells in vivo and in vitro: Relevance for antitumor activity?. Urol. Res..

[27] Bisiaux A., Thiounn N., Timsit M.-O., Eladaoui A., Chang H.-H., Mapes J., Mogenet A., Bresson J.-L., Prié D., Béchet S. (2009). Molecular analyte profiling of the early events and tissue conditioning following intravesical bacillus calmette-guerin therapy in patients with superficial bladder cancer. J. Urol..

[28] Ludwig A.T., Moore J.M., Luo Y., Chen X., Saltsgaver N.A., O’Donnell M.A., Griffith T.S. (2004). Tumor necrosis factor-related apoptosis-inducing ligand: A novel mechanism for Bacillus Calmette-Guérin-induced antitumor activity. Cancer Res..

[29] Lage J.M., Bauer W.C., Kelley D.R., Ratliff T.L., Catalona W.J. (1986). Histological parameters and pitfalls in the interpretation of bladder biopsies in bacillus Calmette-Guerin treatment of superficial bladder cancer. J. Urol..

[30] Mitropoulos D.N. (2005). Novel insights into the mechanism of action of intravesical immunomodulators. In Vivo.

[31] Stefanini G.F., Bercovich E., Mazzeo V., Grigioni W.F., Emili E., D’Errico A., Lo Cigno M., Tamagnini N., Mazzetti M. (1989). Class I and class II HLA antigen expression by transitional cell carcinoma of the bladder: Correlation with T-cell infiltration and BCG treatment. J. Urol..

[32] Ikeda N., Toida I., Iwasaki A., Kawai K., Akaza H. (2002). Surface antigen expression on bladder tumor cells induced by bacillus Calmette-Guérin (BCG): A role of BCG internalization into tumor cells. Int. J. Urol..

[33] Luo Y. (2012). Blocking IL-10 enhances bacillus Calmette-Guérin induced T helper Type 1 immune responses and anti-bladder cancer immunity. Oncoimmunology.

[34] Pettenati C., Ingersoll M.A. (2018). Mechanisms of BCG immunotherapy and its outlook for bladder cancer. Nat. Rev. Urol..

[35] Pardoll D.M. (2012). The blockade of immune checkpoints in cancer immunotherapy. Nat. Rev. Cancer.

[36] Topalian S.L., Taube J.M., Anders R.A., Pardoll D.M. (2016). Mechanism-driven biomarkers to guide immune checkpoint blockade in cancer therapy. Nat. Rev. Cancer.

[37] Kates M., Matoso A., Choi W., Baras A.S., Daniels M.J., Lombardo K., Brant A., Mikkilineni N., McConkey D.J., Kamat A.M. (2020). Adaptive immune resistance to intravesical BCG in non–muscle invasive bladder cancer: Implications for prospective BCG-unresponsive trials. Clin. Cancer Res..

[38] Pierconti F., Raspollini M.R., Martini M., Larocca L.M., Bassi P.F., Bientinesi R., Baroni G., Minervini A., Petracco G., Pini G.M. (2020). PD-L1 expression in bladder primary in situ urothelial carcinoma: Evaluation in BCG-unresponsive patients and BCG responders. Virchows Arch..

[39] Hashizume A., Umemoto S., Yokose T., Nakamura Y., Yoshihara M., Shoji K., Wada S., Miyagi Y., Kishida T., Sasada T. (2018). Enhanced expression of PD-L1 in non-muscle-invasive bladder cancer after treatment with Bacillus Calmette-Guerin. Oncotarget.

[40] Fukumoto K., Kikuchi E., Mikami S., Hayakawa N., Matsumoto K., Niwa N., Oya M. (2018). Clinical Role of Programmed Cell Death-1 Expression in Patients with Non-muscle-invasive Bladder Cancer Recurring After Initial Bacillus Calmette–Guérin Therapy. Ann. Surg. Oncol..

[41] Chevalier M.F., Schneider A.K., Cesson V., Dartiguenave F., Lucca I., Jichlinski P., Nardelli-Haefliger D., Derré L. (2018). Conventional and PD-L1-expressing Regulatory T Cells are Enriched During BCG Therapy and may Limit its Efficacy. Eur. Urol..

[42] Copland A., Sparrow A., Hart P., Diogo G.R., Paul M., Azuma M., Reljic R. (2019). Bacillus Calmette-Guérin Induces PD-L1 Expression on Antigen-Presenting Cells via Autocrine and Paracrine Interleukin-STAT3 Circuits. Sci. Rep..

[43] Alexandrov L.B., Nik-Zainal S., Wedge D.C., Aparicio S.A.J.R., Behjati S., Biankin A.V., Bignell G.R., Bolli N., Borg A., Børresen-Dale A.L. (2013). Signatures of mutational processes in human cancer. Nature.

[44] Samstein R.M., Lee C.-H., Shoushtari A.N., Hellmann M.D., Shen R., Janjigian Y.Y., Barron D.A., Zehir A., Jordan E.J., Omuro A. (2019). Tumor mutational load predicts survival after immunotherapy across multiple cancer types. Nat. Genet..

[45] Bellmunt J., de Wit R., Vaughn D.J., Fradet Y., Lee J.-L., Fong L., Vogelzang N.J., Climent M.A., Petrylak D.P., Choueiri T.K. (2017). Pembrolizumab as Second-Line Therapy for Advanced Urothelial Carcinoma. N. Engl. J. Med..

[46] Roviello G., Catalano M., Santi R., Palmieri V.E., Vannini G., Galli I.C., Buttitta E., Villari D., Rossi V., Nesi G. (2021). Immune checkpoint inhibitors in urothelial bladder cancer: State of the art and future perspectives. Cancers.

[47] Powles T., Park S.H., Voog E., Caserta C., Valderrama B.P., Gurney H., Kalofonos H., Radulović S., Demey W., Ullén A. (2020). Avelumab Maintenance Therapy for Advanced or Metastatic Urothelial Carcinoma. N. Engl. J. Med..

[48] Bajorin D.F., Witjes J.A., Gschwend J.E., Schenker M., Valderrama B.P., Tomita Y., Bamias A., Lebret T., Shariat S.F., Park S.H. (2021). Adjuvant Nivolumab versus Placebo in Muscle-Invasive Urothelial Carcinoma. N. Engl. J. Med..

[49] Rosenberg J.E., Hoffman-Censits J., Powles T., van der Heijden M.S., Balar A.V., Necchi A., Dawson N., O’Donnell P.H., Balmanoukian A., Loriot Y. (2016). Atezolizumab in patients with locally advanced and metastatic urothelial carcinoma who have progressed following treatment with platinum-based chemotherapy: A single-arm, multicentre, phase 2 trial. Lancet.

[50] Black P.C., Tangen C., Singh P., McConkey D.J., Lucia S., Lowrance W.T., Koshkin V.S., Stratton K.L., Bivalacqua T., Sharon E. (2020). Phase II trial of atezolizumab in BCG-unresponsive non-muscle invasive bladder cancer: SWOG S1605 (NCT #02844816). J. Clin. Oncol..

[51] Michael C., Abhishek T., Sanjay P., Daniel Z., Yuejin W., Riza F., Kelly S. (2021). LBA02-04 Novel weekly immunotherapy dosing with avelumab tolerated during Bacillus Calmette-Guerin induction therapy: Initial results of the ABC trial. J. Urol..

[52] Definition of Multipeptide Vaccine S-588210—NCI Drug Dictionary—National Cancer Institute. https://www.cancer.gov/publications/dictionaries/cancer-drug/def/multipeptide-vaccine-s-588210.

[53] Duplisea J.J., Mokkapati S., Plote D., Schluns K.S., McConkey D.J., Yla-Herttuala S., Parker N.R., Dinney C.P. (2019). The development of interferon-based gene therapy for BCG unresponsive bladder cancer: From bench to bedside. World J. Urol..

[54] Malmström P.-U. (2002). A randomized comparative dose-ranging study of interferon-alpha and mitomycin-C as an internal control in primary or recurrent superficial transitional cell carcinoma of the bladder. BJU Int..

[55] Connor R.J., Anderson J.M., Machemer T., Maneval D.C., Engler H. (2005). Sustained intravesical interferon protein exposure is achieved using an adenoviral-mediated gene delivery system: A study in rats evaluating dosing regimens. Urology.

[56] Dinney C.P.N., Fisher M.B., Navai N., O’Donnell M.A., Cutler D., Abraham A., Young S., Hutchins B., Caceres M., Kishnani N. (2013). Phase I trial of intravesical recombinant adenovirus mediated interferon-α2b formulated in Syn3 for Bacillus Calmette-Guérin failures in nonmuscle invasive bladder cancer. J. Urol..

[57] Shore N.D., Boorjian S.A., Canter D.J., Ogan K., Karsh L.I., Downs T.M., Gomella L.G., Kamat A.M., Lotan Y., Svatek R.S. (2017). Intravesical rAd-IFNα/Syn3 for Patients With High-Grade, Bacillus Calmette-Guerin-Refractory or Relapsed Non-Muscle-Invasive Bladder Cancer: A Phase II Randomized Study. J. Clin. Oncol..

[58] Schuckman A.K., Lotan Y., Boorjian S.A., Cilwa K.E., Dinney C.P.N. (2021). MP16-01 Efficacy of nadofaragene firadenovec for patients with carcinoma in situ (CIS), BCG-unresponsive non-muscle invasive bladder cancer (NMIBC): Longer-term follow-up from the phase III trial. J. Urol..

[59] Lotan Y., Schuckman A.K., Angeles L., Boorjian S.A., Cilwa K.E., Dinney C.P.N. (2021). MP16-02 Phase III trial of intravescical nadofaragene firadenovec in patients with high-grade BCG-unresponsive, non-muscle invasive bladder cancer: Two-year follow up in the Ta/T1 cohort. J. Urol..

[60] Ramesh N., Ge Y., Ennist D.L., Zhu M., Mina M., Ganesh S., Reddy P.S., Yu D.-C. (2006). CG0070, a Conditionally Replicating Granulocyte Macrophage Colony-Stimulating Factor–Armed Oncolytic Adenovirus for the Treatment of Bladder Cancer. Clin. Cancer Res..

[61] Packiam V.T., Lamm D.L., Barocas D.A., Trainer A., Fand B., Davis R.L., Clark W., Kroeger M., Dumbadze I., Chamie K. (2018). An open label, single-arm, phase II multicenter study of the safety and efficacy of CG0070 oncolytic vector regimen in patients with BCG-unresponsive non–muscle-invasive bladder cancer: Interim results. Urol. Oncol. Semin. Orig. Investig..

[62] Cheong J.E., Ekkati A., Sun L. (2018). A patent review of IDO1 inhibitors for cancer. Expert Opin. Ther. Pat..

[63] Chu C.E., Porten S.P., Grossfeld G.D., Meng M.V. (2020). Role of Indoleamine-2,3-Dioxygenase Inhibitors in Salvage Therapy for Non-Muscle Invasive Bladder Cancer. Urol. Clin. N. Am..

[64] Waldmann T.A. (2006). The biology of interleukin-2 and interleukin-15: Implications for cancer therapy and vaccine design. Nat. Rev. Immunol..

[65] Gomes-Giacoia E., Miyake M., Goodison S., Sriharan A., Zhang G., You L., Egan J.O., Rhode P.R., Parker A.S., Chai K.X. (2014). Intravesical ALT-803 and BCG treatment reduces tumor burden in a carcinogen induced bladder cancer rat model; a role for cytokine production and NK cell expansion. PLoS ONE.

[66] Rosser C.J., Tikhonenkov S., Nix J.W., Chan O.T.M., Ianculescu I., Reddy S., Soon-Shiong P. (2021). Safety, Tolerability, and Long-Term Clinical Outcomes of an IL-15 analogue (N-803) Admixed with Bacillus Calmette-Guérin (BCG) for the Treatment of Bladder Cancer. Oncoimmunology.

[67] Chamie K., Lee J.H., Rock A., Rhode P.R., Soon-Shiong P. (2019). Preliminary phase 2 clinical results of IL-15RαFc superagonist N-803 with BCG in BCG-unresponsive non-muscle invasive bladder cancer (NMIBC) patients. J. Clin. Oncol..

[68] Ohadian Moghadam S., Nowroozi M.R. (2019). Toll-like receptors: The role in bladder cancer development, progression and immunotherapy. Scand. J. Immunol..

[69] Camargo J.A., Passos G.R., Ferrari K.L., Billis A., Saad M.J.A., Reis L.O. (2018). Intravesical Immunomodulatory Imiquimod Enhances Bacillus Calmette-Guérin Downregulation of Nonmuscle-invasive Bladder Cancer. Clin. Genitourin. Cancer.

[70] Donin N.M., Chamie K., Lenis A.T., Pantuck A.J., Reddy M., Kivlin D., Holldack J., Pozzi R., Hakim G., Karsh L.I. (2017). A phase 2 study of TMX-101, intravesical imiquimod, for the treatment of carcinoma in situ bladder cancer. Urol. Oncol. Semin. Orig. Investig..

[71] Rentsch C.A., Thalmann G.N., Lucca I., Kwiatkowski M., Wirth G.J., Strebel R.T., Engeler D., Pedrazzini A., Hüttenbrink C., Schultze-Seemann W. (2022). A Phase 1/2 Single-arm Clinical Trial of Recombinant Bacillus Calmette-Guérin (BCG) VPM1002BC Immunotherapy in Non–muscle-invasive Bladder Cancer Recurrence after Conventional BCG Therapy: SAKK 06/14. Eur. Urol. Oncol..

[72] Huang L., Yang Y., Yang F., Liu S., Zhu Z., Lei Z., Guo J. (2018). Functions of EpCAM in physiological processes and diseases. Int. J. Mol. Med..

[73] Kowalski M., Guindon J., Brazas L., Moore C., Entwistle J., Cizeau J., Jewett M.A.S., MacDonald G.C. (2012). A phase II study of oportuzumab monatox: An immunotoxin therapy for patients with noninvasive urothelial carcinoma in situ previously treated with bacillus Calmette-Guérin. J. Urol..

[74] Heath E.I., Rosenberg J.E. (2021). The biology and rationale of targeting nectin-4 in urothelial carcinoma. Nat. Rev. Urol..

[75] Kardoust Parizi M., Margulis V., Lotan Y., Mori K., Shariat S.F. (2021). Fibroblast growth factor receptor: A systematic review and meta-analysis of prognostic value and therapeutic options in patients with urothelial bladder carcinoma. Urol. Oncol. Semin. Orig. Investig..

[76] Hahn N.M., Bivalacqua T.J., Ross A., Netto G.J., Park J.C., Masterson T.A., Koch M.O., Bihrle R., Foster R., Gardner T.A. (2016). Phase 2 trial of dovitinib in Bacillus Calmette-Guerin (BCG) refractory urothelial carcinoma (UC) with tumor FGFR3 mutations or over-expression: Hoosier Cancer Research Network GU12-157. J. Clin. Oncol..

[77] Yin S.-Q., Wang J.-J., Zhang C.-M., Liu Z.-P. (2012). The Development of MetAP-2 Inhibitors in Cancer Treatment. Curr. Med. Chem..

[78] Ye D., Yao X., Wang G., Pu J., Yao X., Zhou F., Qi J., Ye Z., Xie L., Chen J. (2017). An oral methionine aminopeptidase II inhibitor for high-risk non-muscle invasive bladder cancer relapsed after intravesical therapies: Update of a phase II trial. J. Clin. Oncol..

[79] Pinto-Leite R., Arantes-Rodrigues R., Sousa N., Oliveira P.A., Santos L. (2016). mTOR inhibitors in urinary bladder cancer. Tumor Biol..

[80] Álvarez-Maestro M., Guerrero-Ramos F., Rodríguez-Faba O., Domínguez-Escrig J., Fernández-Gómez J. (2021). Current treatments for BCG failure in non-muscle invasive bladder cancer (NMIBC). Actas Urológicas Españolas.

[81] Nativ O., Witjes J.A., Hendricksen K., Cohen M., Kedar D., Sidi A., Colombo R., Leibovitch I. (2009). Combined Thermo-Chemotherapy for Recurrent Bladder Cancer After Bacillus Calmette-Guerin. J. Urol..

[82] De Jong J.J., Hendricksen K., Rosier M., Mostafid H., Boormans J.L. (2018). Hyperthermic Intravesical Chemotherapy for BCG Unresponsive Non-Muscle Invasive Bladder Cancer Patients. Bladder Cancer.

[83] Soria F., Milla P., Fiorito C., Pisano F., Sogni F., Di Marco M., Pagliarulo V., Dosio F., Gontero P. (2016). Efficacy and safety of a new device for intravesical thermochemotherapy in non-grade 3 BCG recurrent NMIBC: A phase I–II study. World J. Urol..

[84] Racioppi M., DI Gianfrancesco L., Ragonese M., Palermo G., Sacco E., Bassi P.F. (2018). ElectroMotive drug administration (EMDA) of Mitomycin C as first-line salvage therapy in high risk “bCG failure” non muscle invasive bladder cancer: 3 years follow-up outcomes. BMC Cancer.

